# Supplementary material for: Research of the mechanism on miRNA193 in exosomes promotes cisplatin resistance in esophageal cancer cells
Source: PLoS One. 2020 May 5;15(5):e0225290. doi: 10.1371/journal.pone.0225290 (PMC7199973; doi:10.1371/journal.pone.0225290)

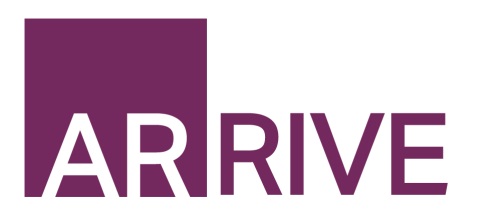


The ARRIVE Guidelines Checklist

Animal Research: Reporting In Vivo Experiments

Carol Kilkenny^1^, William J Browne^2^, Innes C Cuthill^3^, Michael Emerson^4^ and Douglas G Altman^5^

*^1^The National Centre for the Replacement, Refinement and Reduction of Animals in Research, London, UK, ^2^School of Veterinary Science, University of Bristol, Bristol, UK, ^3^School of Biological Sciences, University of Bristol, Bristol, UK, ^4^National Heart and Lung Institute, Imperial College London, UK, ^5^Centre for Statistics in Medicine, University of Oxford, Oxford, UK.*

|  | | ITEM | RECOMMENDATION | Section/ Paragraph |
| --- | --- | --- | --- | --- |
| 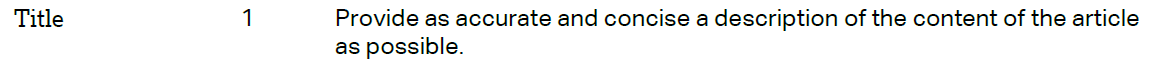 | | | Title |  |
| 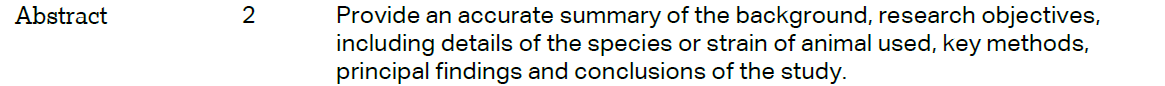 | | | Abstract  Paragraph 1-4 |  |
| INTRODUCTION | | |  |  |
| 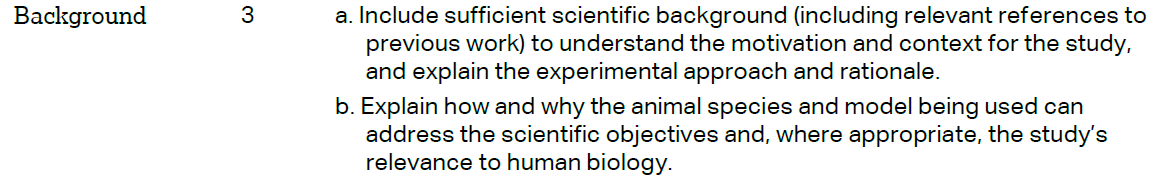 | | | Introduction Paragraph 1-3 |  |
| 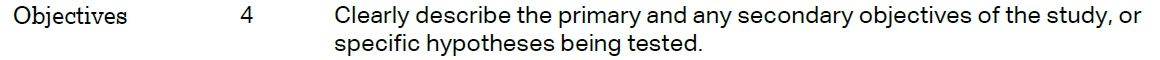 | | | Abstract  Paragraph 1 |  |
| METHODS | | |  |  |
| 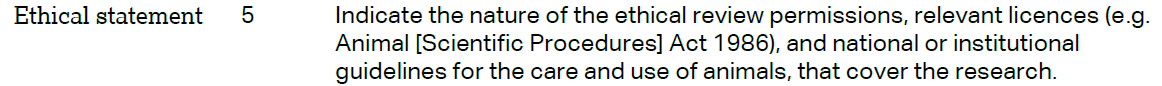 | | | Materials and methods Paragraph 17 |  |
| 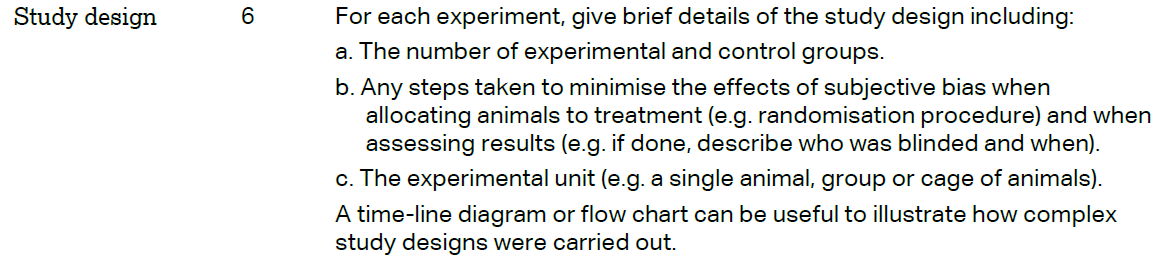 | | | Materials and methods Paragraph 17 |  |
| 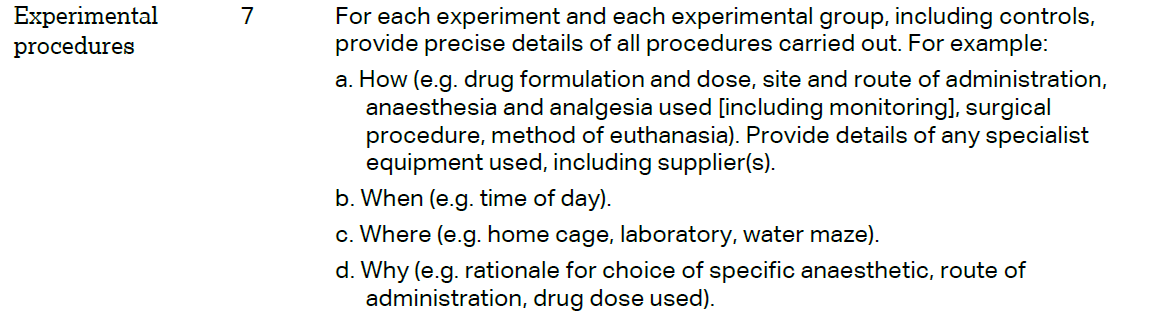 | | | Materials and methods Paragraph 17 |  |
| 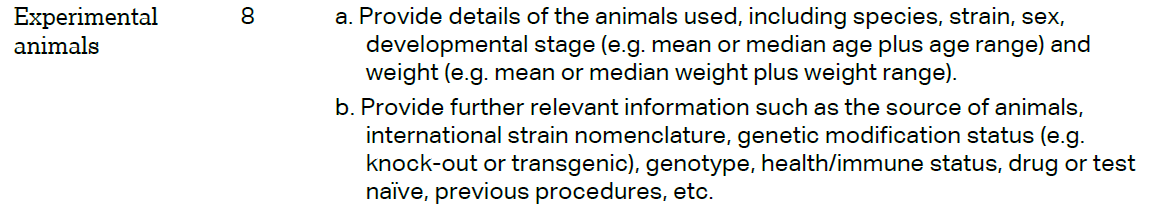 | | | Materials and methods Paragraph 17 |  |

The ARRIVE guidelines. Originally published in *PLoS Biology*, June 2010^1^

| 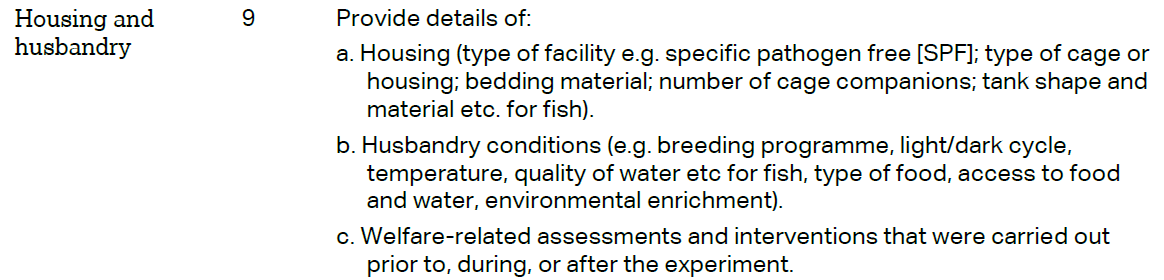 | Materials and methods Paragraph 17 | |
| --- | --- | --- |
| 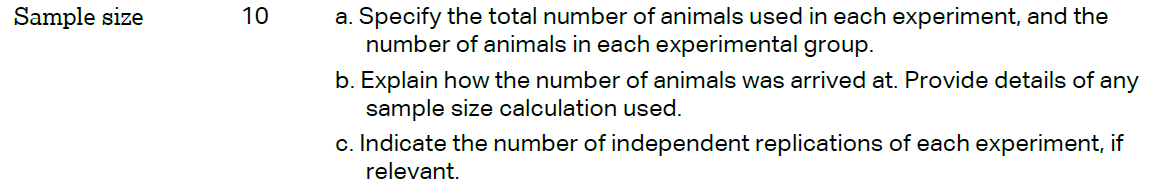 | Materials and methods Paragraph 17 | |
| 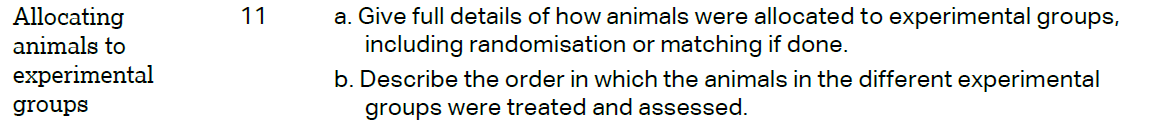 | Materials and methods Paragraph 17 | |
| 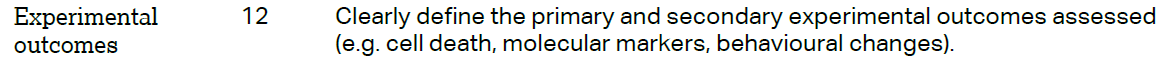 |  | |
| 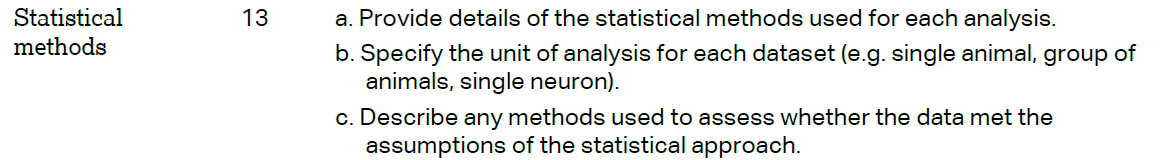 | Materials and methods Paragraph 18 | |
| RESULTS |  | |
| 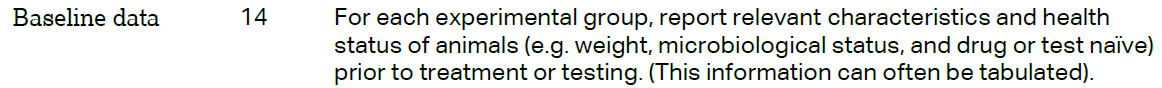 | Not applicable | |
| 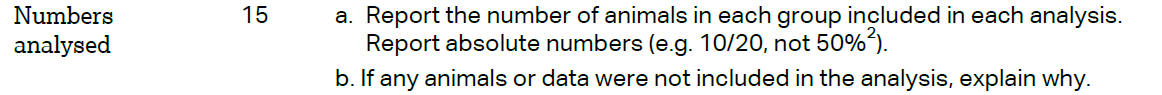 | Not applicable | |
| 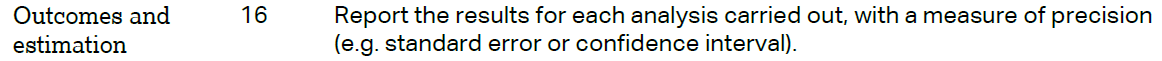 | Results Paragraph13 | |
| 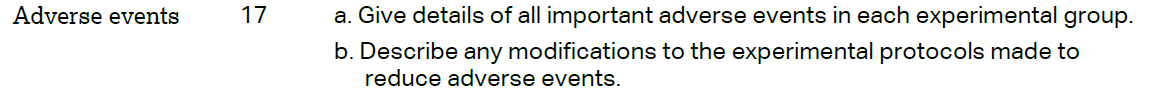 | Not applicable | |
| DISCUSSION |  | |
| 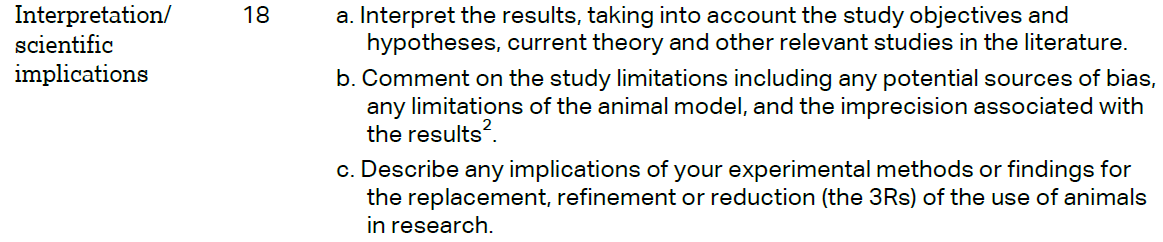 | Not applicable | |
| 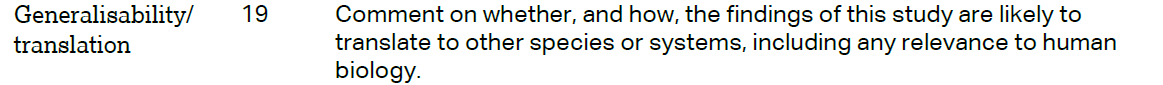 | Not applicable | |
| 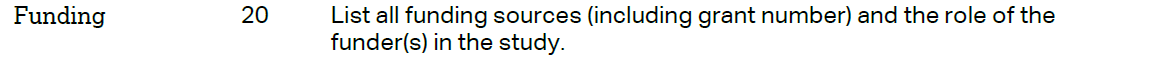 | | Not applicable |


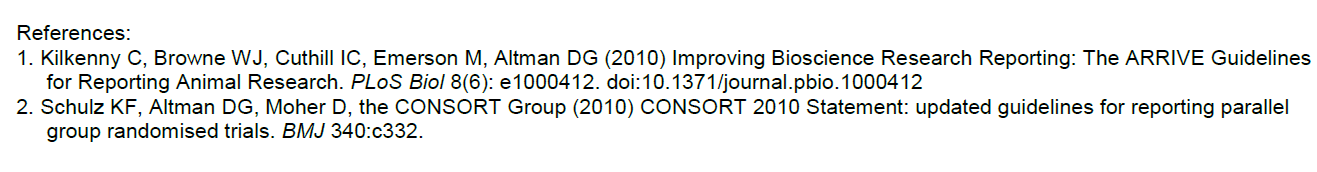

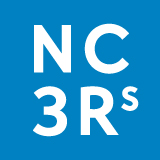

Supplement: S1 Checklist — (DOCX) [file pone.0225290.s001.docx]
