# Supplementary figures and images for: Research of the mechanism on miRNA193 in exosomes promotes cisplatin resistance in esophageal cancer cells
Source: PLoS One. 2020 May 5;15(5):e0225290. doi: 10.1371/journal.pone.0225290 (PMC7199973; doi:10.1371/journal.pone.0225290)

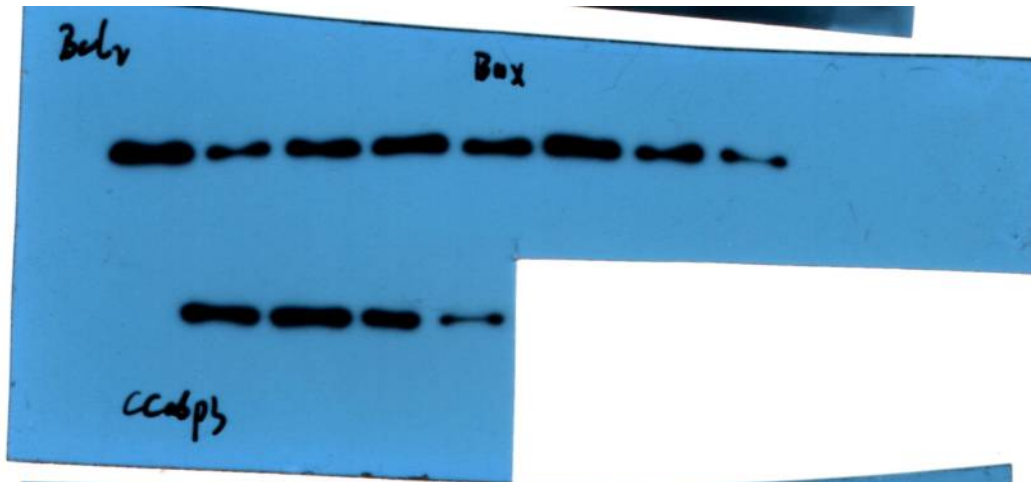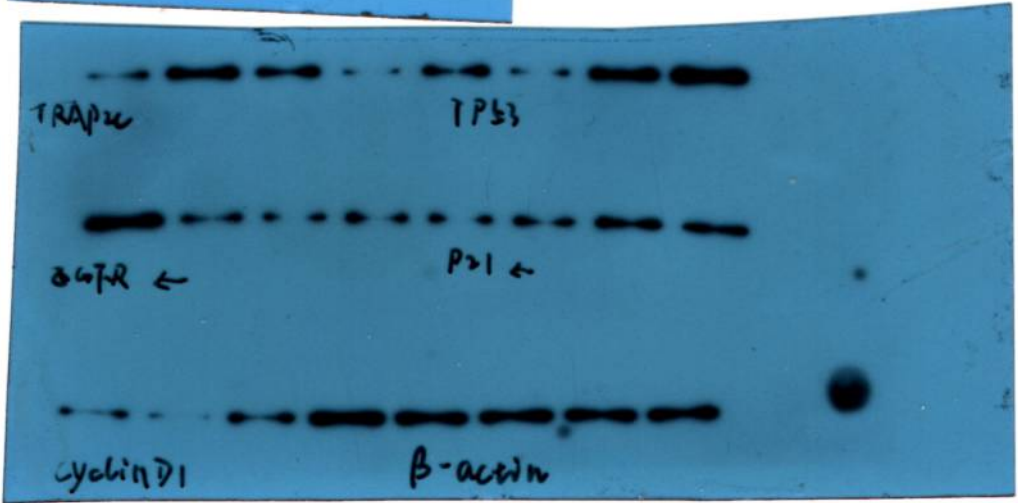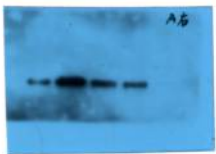

TFAP2C

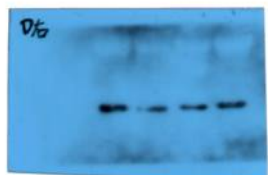

Bcl2

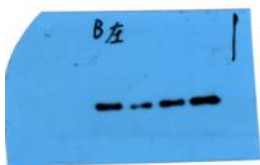

TP53

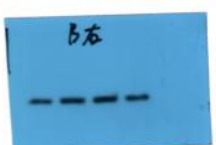

P21

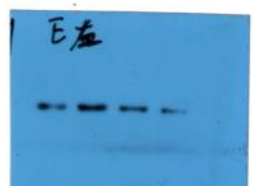

Bax

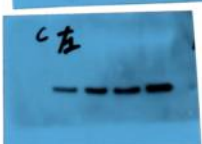

EGFR

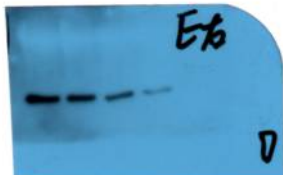

Casp3

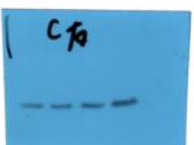

CyclinD1

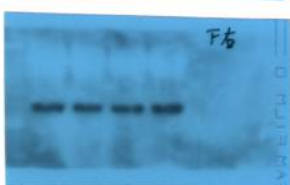

Actin

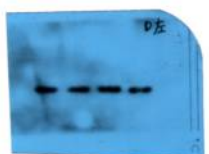

Actin

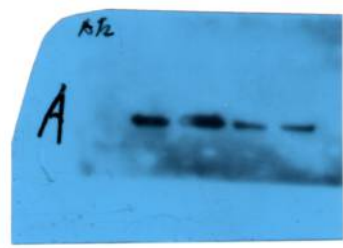

CD63

Supplement: S1 Raw Image — (PDF) [file pone.0225290.s002.pdf]
