# Supplementary material for: Research of the mechanism on miRNA193 in exosomes promotes cisplatin resistance in esophageal cancer cells
Source: PLoS One. 2020 May 5;15(5):e0225290. doi: 10.1371/journal.pone.0225290 (PMC7199973; doi:10.1371/journal.pone.0225290)
Supplement: S1 Table — (DOCX) [file pone.0225290.s003.docx]

**Supplementary Table 1** qPCR primer sequences

| qPCR primer sequences | | |
| --- | --- | --- |
| Gene | Sequence 5'→3' | Size(bp) |
| miRNA-193 | TGGCCTACAAAGTCCCAGTAAA |  |
| Bax-F | AAGAAGCTGAGCGAGTGT | 78 |
| Bax-R | GGCGGCAATCATCCTCTG |  |
| BCL2-F | ACATCCTATCAACAACAA | 135 |
| BCL2-R | GTATCTACACTACAGTCTTA |  |
| Caspase3-F | GACATACTCCTTCCATCAA | 183 |
| Caspase3-R | ATTCATAGCACAGCATCA |  |
| EGFR-F | TTGTTGTGTCTGGTTGTT | 90 |
| EGFR-R | GATGTGTATGTCCGTGATT |  |
| P21-F | GGATAATGGTGATTGAGAT | 76 |
| P21-R | ATCATCTTCATGGCTTTG |  |
| TFAP2C-F | CTCTCCTGACATCCTTAGTAG | 121 |
| TFAP2C-R | ATTCGGCTTCACAGACATA |  |
| TP53-F | TTACAATCAGCCACATTC | 92 |
| TP53-R | TTACAATCAGCCACATTC |  |
| GAPDH-F | CTCTGGTAAAGTGGATATTGT | 85 |
| GAPDH-R | GGTGGAATCATATTGGAACA |  |
